# Cellular Mechanism of the Nonmonotonic Dose Response of Bisphenol A in Rat Cardiac Myocytes

**DOI:** 10.1289/ehp.1307491

**Published:** 2014-02-25

**Authors:** Qian Liang, Xiaoqian Gao, Yamei Chen, Kui Hong, Hong-Sheng Wang

**Affiliations:** 1 Department of Cardiology, Second Affiliated Hospital of Nanchang University, Nanchang, China; 2 Department of Pharmacology, University of Cincinnati College of Medicine, Cincinnati, Ohio, USA

## Abstract

Background: The need for mechanistic understanding of nonmonotonic dose responses has been identified as one of the major data gaps in the study of bisphenol A (BPA). Previously we reported that acute exposure to BPA promotes arrhythmogenesis in female hearts through alteration of myocyte Ca^2+^ handling, and that the dose response of BPA was inverted U-shaped.

Objective: We sought to define the cellular mechanism underlying the nonmonotonic dose response of BPA in the heart.

Methods: We examined rapid effects of BPA in female rat ventricular myocytes using video-edge detection, confocal and conventional fluorescence imaging, and patch clamp.

Results: The rapid effects of BPA in cardiac myocytes, as measured by multiple end points, including development of arrhythmic activities, myocyte mechanics, and Ca^2+^ transient, were characterized by nonmonotonic dose responses. Interestingly, the effects of BPA on individual processes of myocyte Ca^2+^ handling were monotonic. Over the concentration range of 10^–12^ to 10^–6^ M, BPA progressively increased sarcoplasmic reticulum (SR) Ca^2+^ release and Ca^2+^ reuptake and inhibited the L-type Ca^2+^ current (I_CaL_). These effects on myocyte Ca^2+^ handling were mediated by estrogen receptor (ER) β signaling. The nonmonotonic dose responses of BPA can be accounted for by the combined effects of progressively increased SR Ca^2+^ reuptake/release and decreased Ca^2+^ influx through I_CaL_.

Conclusion: The rapid effects of BPA on female rat cardiac myocytes are characterized by nonmonotonic dose responses as measured by multiple end points. The nonmonotonic dose response was produced by ERβ-mediated monotonic effects on multiple cellular Ca^2+^ handling processes. This represents a distinct mechanism underlying the nonmonotonicity of BPA’s actions.

Citation: Liang Q, Gao X, Chen Y, Hong K, Wang HS. 2014. Cellular mechanism of the nonmonotonic dose response of bisphenol A in rat cardiac myocytes. Environ Health Perspect 122:601–608; http://dx.doi.org/10.1289/ehp.1307491

## Introduction

Bisphenol A (BPA; CAS 80-05-7) is one of the highest production volume chemicals worldwide, with annual production exceeding 3 million metric tons. BPA is used in the production of polycarbonate plastics, epoxy resins, and nonpolymer additives to other plastics. It is used extensively in the manufacturing of common consumer products and goods such as food containers, metal cans (as a protective coating), beverage and baby bottles, receipt paper, and water pipes (Vandenberg et al. 2007). Human exposure to BPA from diet, inhalation, and other exposure routes has been well documented (Geens et al. 2012; Vandenberg et al. 2007). BPA has been detected in > 90% of individuals in various sample populations (Vandenberg et al. 2010).

BPA is an estrogenic endocrine-disrupting chemical (EDC) with potentially adverse impacts on human health (Diamanti-Kandarakis et al. 2009). A large body of evidence has linked BPA exposure to abnormalities such as obesity, diabetes, and disorders of the reproductive and immune systems. Growing evidence also suggests that BPA may have adverse impacts on the cardiovascular system. Several epidemiological studies have reported an associaton between higher human BPA exposure and cardiovascular diseases, including coronary and peripheral arterial diseases (Lang et al. 2008; Melzer et al. 2010, 2012; Shankar et al. 2012). Recently, we reported that acute exposure to environmentally relevant low doses of BPA promoted arrhythmogenic-triggered activities in cardiac myocytes from female rodent hearts (Belcher et al. 2012; Yan et al. 2011). The proarrhythmic action of BPA is manifested as increased frequency of ventricular arrhythmias under stress conditions (Yan et al. 2011), as well as increased duration and severity of ventricular arrhythmias following ischemic injury in female rodent hearts (Yan et al. 2013). We found that alterations of myocyte Ca^2+^(calcium ion) handling, including elevated sarcoplasmic reticulum (SR) Ca^2+^ spontaneous release (or Ca^2+^ leak) and SR Ca^2+^ reuptake, are a key mechanism underlying the proarrhythmic action of BPA (Gao et al. 2013; Yan et al. 2011). These findings point to the potential cardiovascular toxicity of BPA.

Previously we reported that the rapid effect of BPA on the contractility of female cardiac myocytes is characterized by a nonmonotonic dose response (Belcher et al. 2012). A nonmonotonic dose–response curve is one that has a point of inflection where the curve slope switches sign from positive to negative or vice versa. Numerous examples of nonmonotonic dose responses have been reported for a range of EDCs and hormones at the gene expression, cell, tissue/organ, animal, and population levels (see Vandenberg et al. 2012 for a comprehensive review). This pharmacodynamic property is of critical importance to the assessment of toxicity of BPA and other EDCs (Fagin 2012; Myers et al. 2009). The Chapel Hill BPA expert panel identified the need for investigation of the mechanisms underlying nonmonotonic dose responses as one of the major data gaps in BPA research (Wetherill et al. 2007). In the present study, we further define the dose–response properties of BPA in the heart, and elucidate the underlying mechanism of the nonmonotonicity of BPA’s cardiac impact.

## Materials and Methods

*Reagents*. All reagents and solvents used were of the highest purity available. Aqueous solutions were prepared using BPA-free water (18 MΩ; < 6 ppb total oxidizable organics; Milli-Q Advantage A10 system; Millipore Corp., Billerica, MA, USA). Bisphenol A (BPA) obtained from TCI America (lot 111909; ground by Battelle) was provided by the Division of the National Toxicology Program (DNTP) at the National Institute of Environmental Health Sciences. Methyl-piperidino-pyrazole,1,3-bis(4-hydroxyphenyl)-4-methyl-5-[4-(2-piperidinylethoxy)phenol]-1H-pyrazole dihydrochloride (MPP) and 4-[2-phenyl-5,7-bis(trifluoromethyl)pyrazolo[1,5-a]pyrimidin-3-yl]phenol (PHTPP) were obtained from Tocris Cookson (Ellisville, MO, USA), and nifedipine was purchased from Sigma-Aldrich (St. Louis, MO, USA). Isopreterenol, nifedipine, and other chemicals were from Sigma-Aldrich unless otherwise stated.

*Animals.* Animal procedures were performed as previously described by Yan et al. (2013) and in accordance with protocols approved by the University of Cincinnati Institutional Animal Care and Use Committee. The animals were treated humanely and with regard for alleviation of suffering. Adult female Sprague-Dawley rats (200–250 g; Charles River, Spencerville, OH, USA) were housed two per cage in standard polycarbonate caging containing Sani-chip bedding (Irradiated Aspen Sani-chip; P.J. Murphy Forest Products Corp., Montville, NJ, USA) to eliminate possible corn-based mycoestrogen exposure. Room conditions included a 14-hr-light/10-hr-dark cycle, with lights on at 0600 hours. All animals were fed *ad libitum* with Teklad 2020 diet (Harlan Laboratories Inc., Indianapolis, IN, USA), which contains no soybean meal, alfalfa, or animal products that may introduce uncontrolled levels of estrogenic compounds. Sterile drinking water was generated by a dedicated water purification system (Millipore Rios 16 with ELIX UV/Progard 2) that reduces oxidizable organics to < 1% of source levels. Drinking water was dispensed from glass water bottles. A total of 70 animals were used in this study.

*Analysis of myocyte mechanics and Ca^2+^ handling*. Ventricular myocytes from female rat hearts were enzymatically dissociated using Langendorff perfusion, as previously described (Yan et al. 2011, 2013). Isolated myocytes were then suspended in 1.0 mm Ca^2+^-Tyrode solution. We analyzed myocyte contraction, after-contraction, and Ca^2+^ transient and spark as previously described (Yan et al. 2011). Briefly, myocytes were excited by field stimulation with 2-msec 1.5× threshold pulses at a rate of 0.5 Hz. Steady-state myocyte shortening was examined using a video-edge detector (Crescent Electronics, Sandy, UT, USA). After-contraction was measured using stimulation of 2 msec 1.5× threshold pulses at a rate of 2 Hz for 8 sec. To measure Ca^2+^ spark, isolated ventricular myocytes were loaded with fluo-4 acetoxymethyl ester (5 μM; Molecular Probes, Eugene, OR, USA) and imaged with a Zeiss LSM 710 inverted confocal microscope (Carl Zeiss Microscopy, LLC, Thornwood, NY, USA) with an excitation wavelength of 488 nM. Signals were measured with line-scan imaging at 3.07-msec intervals, with each line comprising 512 pixels spaced at 0.056 mm. Image processing and data analysis were performed as previously described (Yan et al. 2011). To measure Ca^2+^ transients, fluorescence signals were measured from fluo-4 loaded myocytes using a Nikon TE 2000 microscope and an InCyt Standard PM photometry system (Intracellular Imaging, Cincinnati, OH, USA). Experiments were performed at room temperature (24°C).

*Patch clamp recording of I_CaL_ (L-type Ca^2+^ current)*. The I_CaL_ was recorded at room temperature (24°C) using the whole-cell patch clamp technique, as previously described (Yan et al. 2011). After the membrane was ruptured, cells were clamped at –50 mV for 5 min to allow dialysis of the intracellular solution and stabilization of the Ca^2+^ currents before measurement began. Data collection and analysis were performed using PCLAMP 9 software (Molecular Devices, Sunnyvale, CA, USA).

*Western blotting*. Western blotting experiments were performed as previously described (Gao et al. 2013). Briefly, isolated female ventricular myocytes were treated with BPA for the indicated length of time, collecte, and snap-frozen in liquid nitrogen. Proteins were extracted with 1× Cell Lysis Buffer (Cell Signaling Technology, Danvers, MA, USA) supplemented with protease inhibitors and phosphatase inhibitors. Equal amounts of protein samples from each treatment group were separated by SDS-PAGE and transferred to a nitrocellulose membrane (Bio-Rad, Hercules, CA, USA). The membrane was then blocked with 5% non-fat milk in phosphate-buffered saline–0.1% Tween, followed by incubation with primary and secondary antibodies. We used ECL™ Western Blotting Analysis System (GE Healthcare, Buckinghamshire, UK) for developing the membrane. We used the following antibodies: anti-phospholamban phospho threonine-17 (pThr17-PLN) and anti-phospholamban (anti-PLN), both from Badrilla (Leeds, UK); and horseradish peroxidase–conjugated anti-mouse and anti-rabbit secondary antbodies (Cell Signaling Technology).

*Statistical analysis*. We conducted statistical analysis using an unpaired *t*-test or by one-way analysis of variance (ANOVA) with differences between treatment groups assessed using a multiple comparison posttest. Frequency of events (e.g., percentage of myocytes with after-contractions) was analyzed using a chi-square test. We considered *p* < 0.05 the minimal level of statistical significance for differences in values. Data were analyzed using SigmaPlot (Systat Software Inc., San Jose, CA, USA) and Excel (Microsoft, Redmond, WA, USA).

## Results

**Figure 1:**
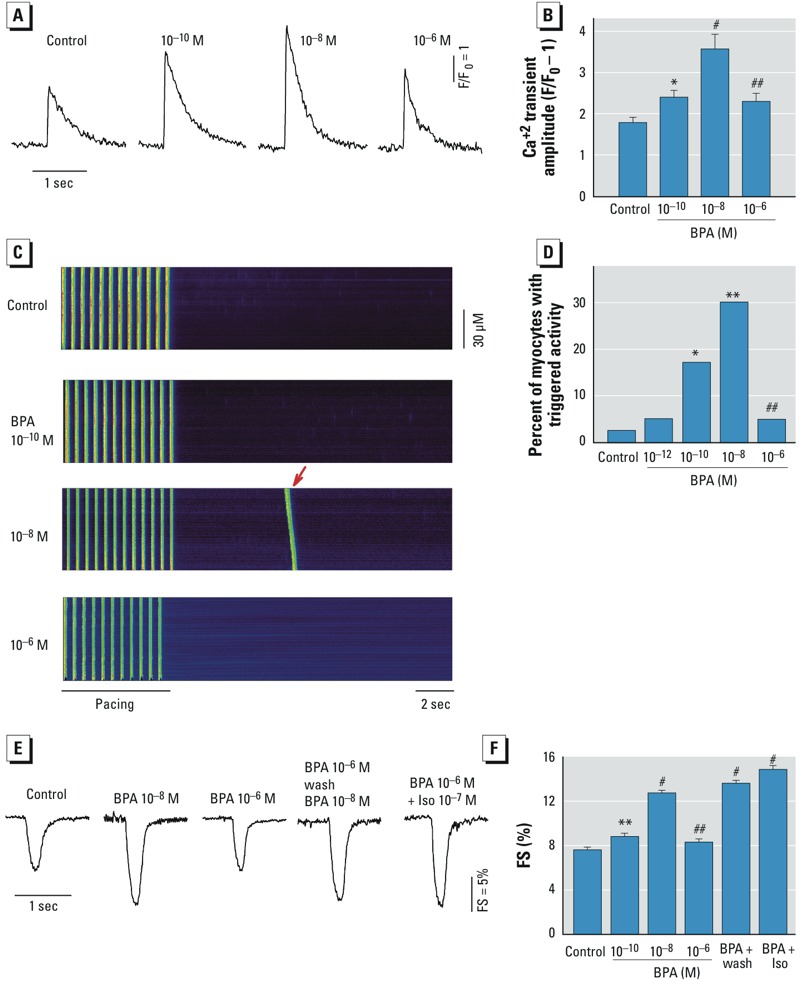
Nonmonotonic dose responses of BPA (10^–10^, 10^–8^,or 10^–6^ M) in female rat ventricular myocytes. F/F_0_, fluorescence intensity ratio. (*A*) Representative Ca^2+^ transient traces after acute exposure to indicated concentrations of BPA or control solution. (*B*) Dose–response of the effects of BPA on myocyte Ca^2+^ transient amplitude (*n* = 23, 18, 17, and 17 myocytes for control and 10^–10^, 10^–8^, and 10^–6^ M BPA, respectively). (*C*) Representative confocal images of Ca^2+^ transients in female rat myocytes elicited by repeated pacing after acute exposure to BPA or the control. Arrows indicate spontaneous Ca^2+^ after-transients (i.e., triggered activity) following pacing. (*D*) Dose–response of the effects of BPA on the percentage of myocytes with triggered activity (*n* = 40, 40, 41, 43, and 41 myocytes for control and 10^–10^, 10^–8^, and 10^–6^ M BPA, respectively). (*E*) Representative contraction traces of myocytes exposed control, BPA, or BPA plus isoproterenol (Iso) as indicated for 2–7 min. (*F*) Dose–response effects of BPA on average fractional shortening (FS) (*n* = 20, 19, 20, 20, 20, and 20 myocytes for control; 10^–10^, 10^–8^, and 10^–6^ M BPA; 10^–6^ M BPA pretreatment + wash; and 10^–6^ M BPA + Iso, respectively). Values shown are mean ± SE. *p < 0.05, **p < 0.01, and ^#^*p *< 0.001, compared with control by one-way ANOVA (*B,F*) or chi-square test (*D*). ^##^*p *< 0.01, compared with 10^–8^ M BPA by unpaired *t*-test or chi-square test.

*Rapid actions of BPA in cardiac myocytes have nonmonotonic dose responses*. We examined the concentration-dependent effect of BPA on Ca^2+^ transient in female rat ventricular myocytes. BPA rapidly (~ 5 min) increased the amplitude of the Ca^2+^ transient at low BPA doses (10^–10^ to 10^–8^ M), and this stimulatory effect diminished at micromolar doses (Figure 1A,B); the dose–response curve was inverted U-shaped with the most efficacious concentration being 10^–8^ M (Figure 1B). Previously we showed that BPA rapidly promoted the development of spontaneous excitation (i.e., triggered activities) in female cardiac myocytes (Yan et al. 2011). The stimulatory effect of BPA on triggered activities was only notable in the nanomolar dose range but not at higher doses (Figure 1C), producing a dose–response curve with pronounced nonmonotonicity (Figure 1D).

One mechanism of the nonmonotonic dose response of hormones and EDCs is general cytotoxicity (Vandenberg et al. 2012); this possibility was examined. Consistent with our previous findings (Belcher et al. 2012), 10^–8^ M BPA rapidly stimulated the contraction of female myocytes, and this stimulatory effect diminished at the micromolar concentration (Figure 1E). Myocytes that were pretreated with 10^–6^ M BPA followed by a wash responded robustly to subsequent exposure to 10^–8^ M BPA (Figure 1F). In addition, the β-adrenergic agonist isoproterenol produced marked stimulatory effect in the presence of 10^–6^ M BPA. These results suggest that the diminished response of myocytes to micromolar concentrations of BPA is not due to nonspecific cytotoxicity of BPA at higher doses.

*BPA alters individual elements of myocyte Ca^2+^ handling with monotonic dose responses*. BPA promotes arrhythmogenesis and enhances myocyte contraction via alteration of myocyte Ca^2+^ handling (Yan et al. 2011). To understand the mechanism of the nonmonotonicity of BPA’s rapid actions in cardiac cells, we examined the dose-dependent impact of BPA on the individual elements of the myocyte Ca^2+^ handling process.

**Figure 2:**
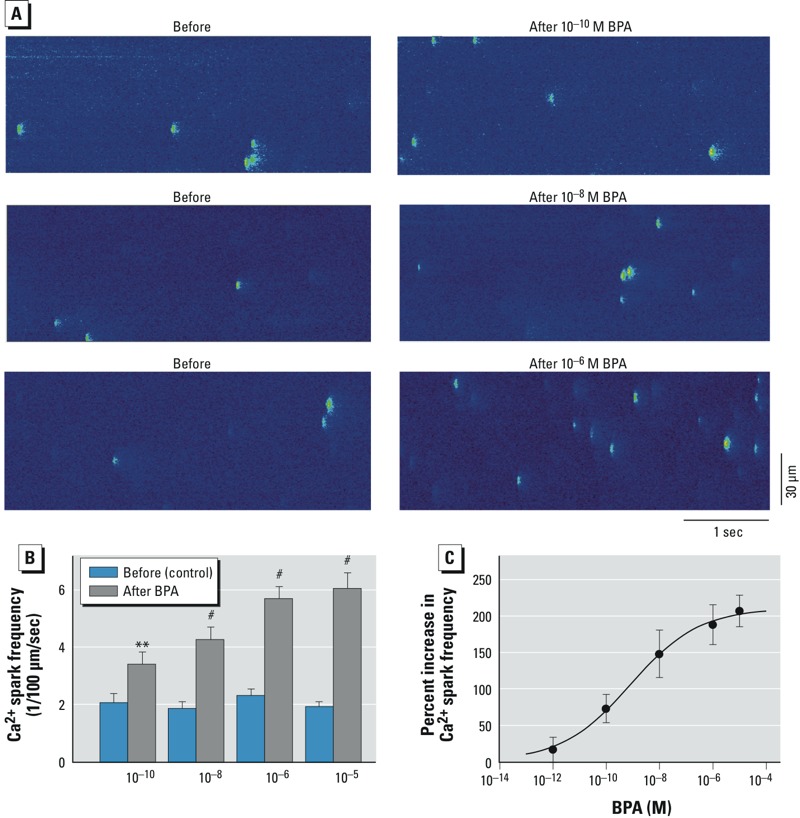
Effect of BPA on SR Ca^2+^ release/leak in female rat ventricular myocytes shows a monotonic dose response. (*A*) Ca^2+^ sparks recorded from three representative myocytes before and after BPA exposure (1–2 min); fluorescent spots indicate local elevations of intracellular Ca^2+^ levels as a result of spontaneous release of Ca^2+^ from the SR (i.e., Ca^2+^ sparks). (*B*) Mean (± SE) spark frequency in myocytes before and after BPA exposure (*n* = 8, 7, 17, and 11 myocytes for 10^–10^, 10^–8^, 10^–6^, and 10^–5^ M BPA, respectively. (*C*) Dose–response curve of BPA’s effect on Ca^2+^ spark frequency. Data are fitted with the Hill equation: Percent increase = maximum increase/{1 + (EC_50_/[BPA])^Hill coefficient^}, where maximum increase = 209.7%, EC_50_ = 0.814 nM, and Hill coefficient = 0.34. **p < 0.01, and ^#^*p *< 0.001, compared with control by paired *t*-test.

*Effect on Ca^2+^ spark*. Increased Ca^2+^ release and Ca^2+^ leak from the SR play a central role in the impact of BPA on arrhythmogenesis and myocyte mechanics (Yan et al. 2011). Diastolic Ca^2+^ release from the SR through the ryanodine receptors was measured as frequency of Ca^2+^ sparks (Cheng et al. 1993). Interestingly, unlike the nonmonotonic dose responses observed at the myocyte level, BPA’s impact on SR Ca^2+^ release was monotonic (Figure 2). Increasing concentrations of BPA over the dose range of 10^–10^ to 10^–5^ M progressively increased Ca^2+^ spark frequency (Figure 2A,B). The dose–response curve had a classic monotonic shape with median effective concentration (EC_50_) of 0.81 nM and a maximum increase of 209.7% (Figure 2C).

**Figure 3:**
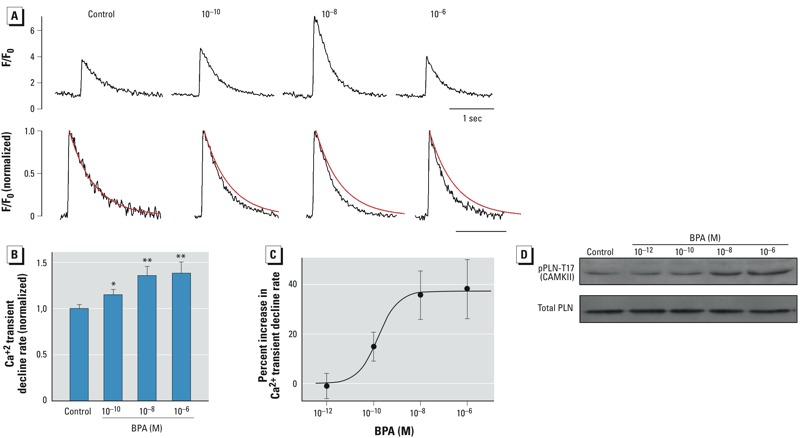
Effect of BPA on SR Ca^2+^ reuptake in female rat ventricular myocytes shows a monotonic dose response. F/F_0_, fluorescence intensity ratio. (*A*) Top, representative Ca^2+^ transient traces after acute exposure to BPA (10^–10^, 10^–8^, or 10^–6^ M) or control solution; bottom, the same traces normalized to an amplitude (F/F_0_) of 1; superimposed red lines represent the single exponential fitting of the decline phase of the Ca^2+^ transient in the control. Rate of Ca^2+^ transient decline (i.e., 1/time constant) indicated SR Ca^2+^ reuptake in rodent cardiac myocytes. (*B*) Mean (± SE) rate of decline (normalized to control); *n* = 23, 18, 17 and 17 myocytes for control and 10^–10^, 10^–8^, and 10^–6^ M BPA, respectively). (*C*) Dose–response curve of BPA’s effect on Ca^2+^ transient decline rate. Data are fitted with the Hill equation: Percent increase = maximum increase/(1 + EC_50_/[BPA]), where maximum increase = 37%, and EC_50_ = 0.15 nM. (*D*) Representative immunoblot showing total PLN and rapid impact of BPA (2‑min exposure) on PLN phosphorylation by CAMKII in female rat myocytes. *p < 0.05, and **p < 0.01, compared with control by one-way ANOVA.

*Effect of BPA on SR Ca^2+^ reuptake*. BPA rapidly increases SR Ca^2+^ reuptake in female rat myocytes (Yan et al. 2011). In rodent cardiac myocytes, SR reuptake accounts for most (> 90%) of Ca^2+^ removal from the cytosol during relaxation (Bers 2002); therefore, the rate of decline of the Ca^2+^ transients can be used as an index of SR Ca^2+^ reuptake. Whereas the effect of BPA on Ca^2+^ transient amplitude was inverted U-shaped (Figure 3A, top), normalization of the Ca^2+^ transient traces revealed that increasing concentrations of BPA progressively increased the rate of decline (Figure 3A, bottom), producing a monotonic dose–response curve (Figure 3B). The dose response had an EC_50_ of 0.15 nM and a maximum increase of 37.3% (Figure 3C).

Through its inhibition of sarco/endoplasmic reticulum Ca^2+^-ATPase (SERCA), PLN is the central regulator of SR Ca^2+^ reuptake. PLN can be phosphorylated by both protein kinase A and Ca^2+^/calmodulin-dependent protein kinase II (CAMKII) at serine 16 and threonine 17, respectively. Phosphorylation of PLN releases its inhibition on SERCA, thereby increasing Ca^2+^ reuptake into the SR (Kranias and Hajjar 2012). Because BPA has been shown to influence SR Ca^2+^ reuptake via increasing CAMKII phosphorylation of PLN at threonine 17 (Gao et al. 2013), we examined the dose-dependent effect of BPA (2-min exposure) on CAMKII phosphorylation of PLN. Increasing concentrations of BPA over the dose range of 10^–12^ M to 10^–6^ M progressively increased phosphorylation of PLN at the CAMKII site (Figure 3D). Based on the known regulatory mechanism of SR Ca^2+^ reuptake, this dose-dependent effect of BPA on PLN phosphorylation should increase SERCA activity, and likely accounts for the monotonic effect of BPA on SR Ca^2+^ reuptake.

**Figure 4:**
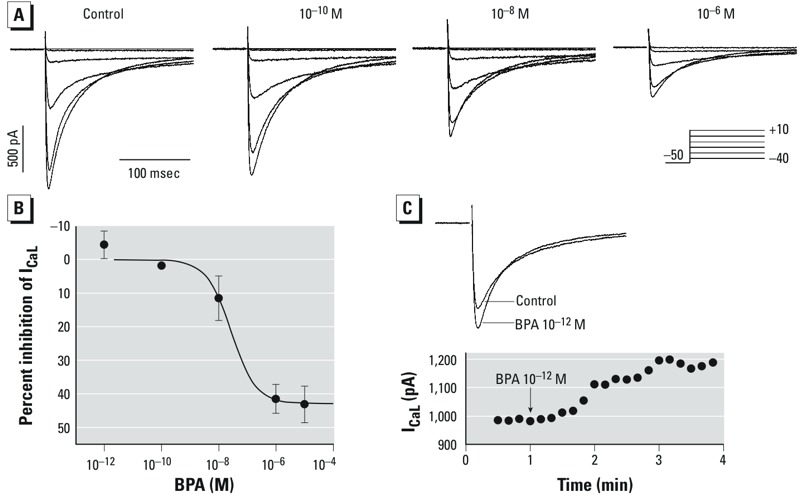
Effect of BPA on the I_CaL_ in female rat ventricular myocytes shows a monotonic dose response. IC_50_, median inhibitory concentration. (*A*) Representative I_CaL_ recorded from the same myocyte before (control) and after exposure to BPA (10^–10^, 10^–8^, or 10^–6^ M). Inset: voltage clamp protocol. (*B*) Dose response of the inhibition of I_CaL_ by BPA; *n* = 7, 5, 7, 4, and 6 for 10^–12^, 10^–10^, 10^–8^, 10^–6^, and 10^–5^ M BPA, respectively. Data are fitted with the Hill equation: Percent inhibition = maximum inhibition/(1 + IC_50_/[BPA]), where maximum inhibition = 43%, and IC_50_ = 27.4 nM. Values are mean ± SE. (*C*) Example of a small but reproducible increase of I_CaL_ after exposure to 10^–12^ M BPA. Top, I_CaL_ recorded at 0 mV; bottom, time course of the observed stimulatory effect. All currents were recorded at steady-state following BPA treatment.

*Effect on I_CaL_*_._ The stimulatory, monotonic effects of BPA on SR Ca^2+^ release and reuptake were countered by an inhibitory action of BPA on the L-type Ca^2+^ current, particularly at higher concentrations (Figure 4A). The dose–response curve for the inhibition of I_CaL_ was, again, monotonic (Figure 4B), with an EC_50_ of 27.4 nM and a maximum inhibition of 43%. The current–voltage relationship of I_CaL_ was not changed by BPA.

Interestingly, although the overall effect of BPA on I_CaL_ was one of suppression, we noticed a small but consistent stimulation of the current at lower BPA concentrations (10^–12^ M) (Figure 4C). The average increase of I_CaL_ at 10^–12^ M BPA was 4.5%, which occurred within minutes of exposure.

**Figure 5:**
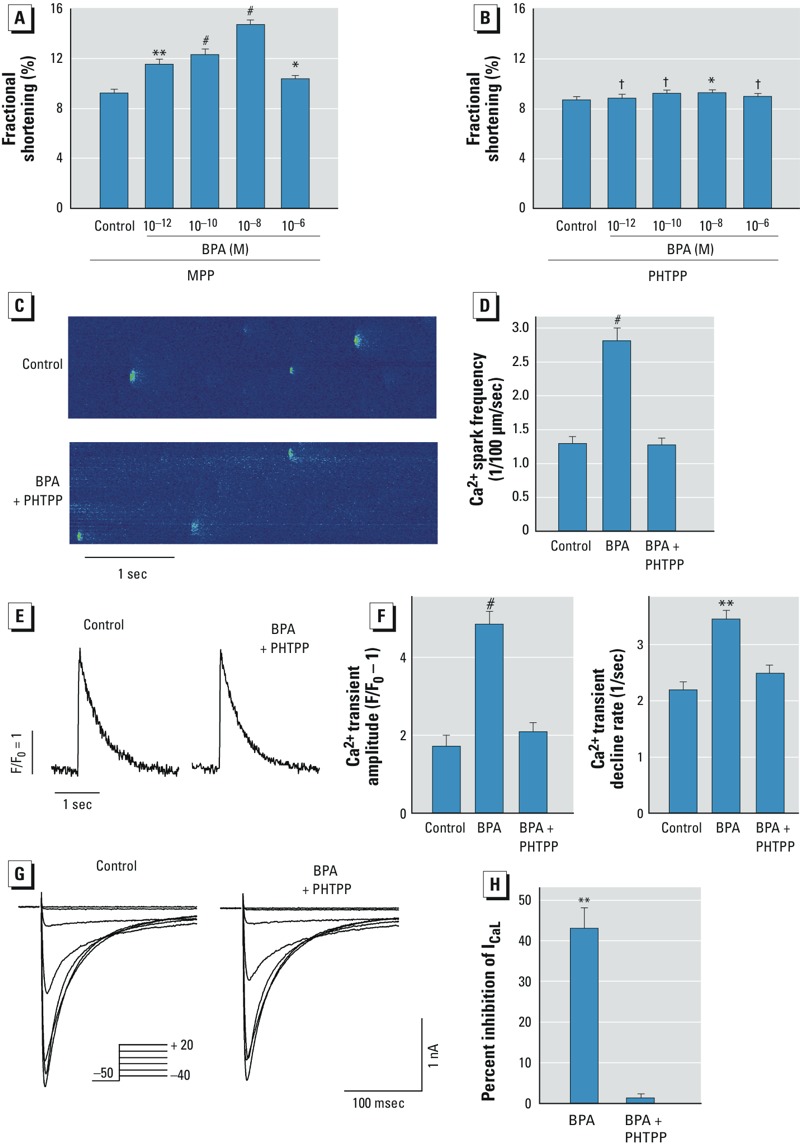
Mediation of the rapid effects of BPA by ERβ signaling in female rat myocytes. F/F_0_, fluorescence intensity ratio. (*A,B*) Effects of the ERα blocker MPP at 10^–6^ M (*A*) and the the ERβ blocker PHTPP at 5 × 10^–6^ M (*B*) on the dose response of BPA on myocyte contractility. For control and 10^–12^, 10^–10^, 10^–8^, and 10^–6^ M BPA, respectively, *n* = 39, 39, 41, 44, and 40 for (*A*), and 24, 24, 21, 24, and 22 for (*B*). (*C*) Ca^2+^ sparks recorded from a representative myocyte before and after exposure to 10^–8^ M BPA (1–2 min) plus PHTPP. (*D*) Average spark frequency in control, 10^–8^ M BPA, and BPA plus PHTPP; *n* = 9, 4, and 5 myocytes, respectively. (*E*) Representative Ca^2+^ transient traces in myocytes treated with control solution or BPA plus PHTPP. (*F*) Average Ca^2+^ transient amplitude (left) and decline rate (right) in control, 10^–8^ M BPA, and BPA plus PHTPP (*n* = 7, 9, and 9 myocytes, respectively. (*G*) Representative I_CaL_ in control myocytes before and after treatment with BPA-plus-PHTPP. Inset: voltage clamp protocol. (*H*) Average inhibition of I_CaL_ by 10^–5^ M BPA or BPA plus PHTPP; *n* = 6 and 3, respectively. Values are mean ± SE. ^†^*p* > 0.1. *p < 0.05, **p < 0.01, and ^#^*p *< 0.001, compared with control by one-way ANOVA.

*BPA’s rapid actions are mediated by ER*β *signaling*. Previously we hypothesized that the opposing actions of estrogen receptor (ER) α and ERβ contribute to the nonmonotonic dose response of BPA in the heart (Belcher et al. 2012). Contrary to this hypothesis, in the presence of MPP, an ERα-selective blocker, the nonmonotonicity of the dose–response curve was unchanged (Figure 5A); ERβ blockade with PHTPP largely abolished the rapid effect of BPA on contractility (Figure 5B).

We also examined the role of ERβ in mediating the effects of BPA on individual Ca^2+^ cycling processes. ERβ blockade with PHTPP completely abolished the stimulatory effect of BPA on Ca^2+^ spark frequency (Figure 5C,D) and blocked effects of BPA on the amplitude and decline kinetics of Ca^2+^ transient (Figure 5E,F). Similarly, PHTPP abolished the inhibitory effect of BPA (10^–5^ M) on I_CaL_ (Figure 5G,H). Thus, ERβ signaling played a dominant role in mediating BPA’s nonmonotonic dose response in cardiac myocytes, as well as in its actions on individual Ca^2+^ cycling processes.

**Figure 6:**
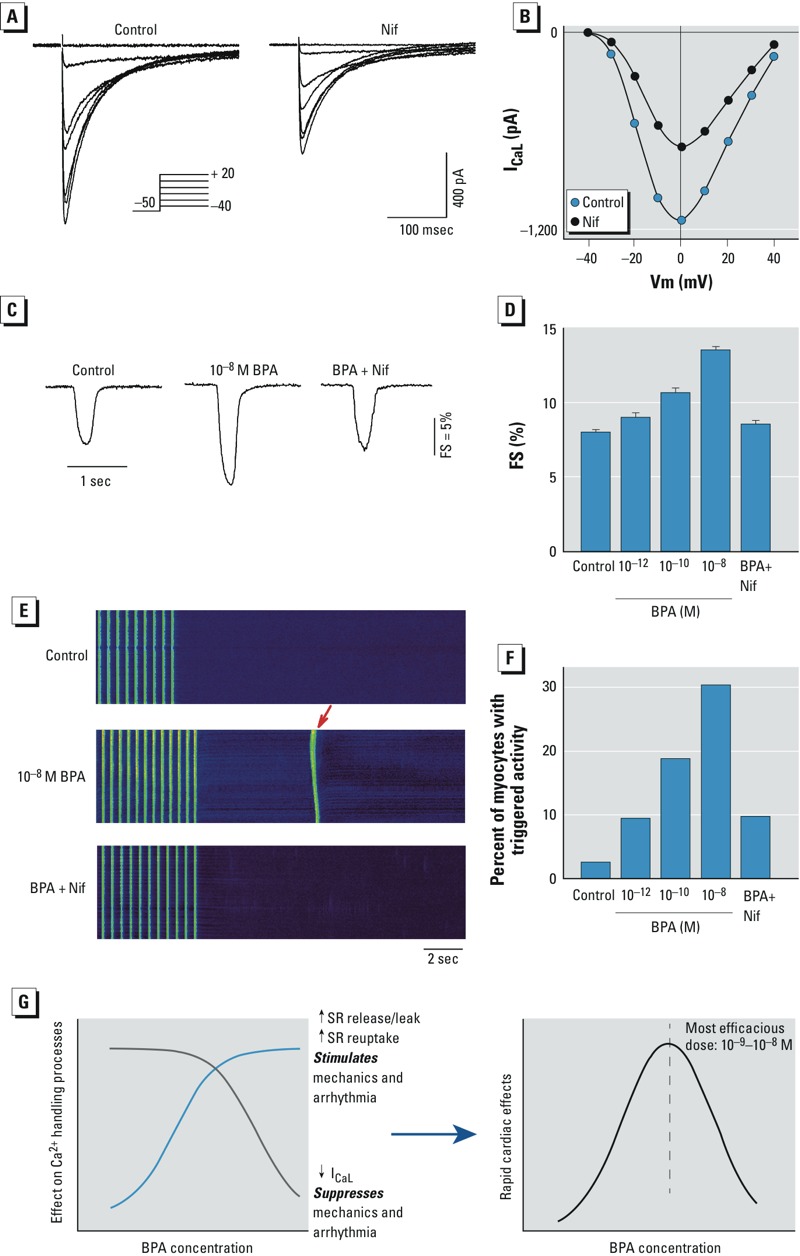
Suppression of I_CaL_ reproduced the nonmonotonic effects of BPA. Current traces (*A*) and current–voltage relationship (*B*) showing the blockade of I_CaL_ by 2 × 10^–7^ M nifedipine (Nif). Inset: voltage clamp protocol. ((*C*) Representative contraction traces in myocytes exposed to control solution, 10^–8^ M BPA, and BPA (10^–8^ M) plus 2 × 10^–7^ M Nif. (*D*) Dose-dependent effects of BPA (10^–12^ to 10^–8^ M) BPA plus Nif on myocyte fractional shortening (FS); *n* = 35, 23, 23, 35, and 37 myocytes for control; 10^–12^, 10^–10^, and 10^–8^ M BPA; and BPA plus Nif, respectively. (*E*) Representative confocal images of Ca^2+^ transients in female rat myocytes elicited by repeated pacing, after acute exposure to control, BPA, and BPA plus Nif; the arrow indicates triggered activity following pacing. (*F*) Dose-dependent effects of BPA and BPA plus Nif on incidence of triggered activity; *n* = 31, 32, 32, 33, 31 myocytes for for control; 10^–12^, 10^–10^, and 10^–8^ M BPA; and BPA plus Nif, respectively. (*G*) Schematic illustration of the cellular mechanism of the nonmonotonic dose response of BPA in female rat ventricular myocytes.

*Suppression of I_CaL_ contributes to the nonmonotonic effects of BPA*. To test the hypothesis that the inhibitory effect of high-dose BPA on I_CaL_ produces the nonmonotonic dose responses, we used the selective I_CaL_ blocker nifedipine to mimic the inhibition by BPA. The stimulatory effects of 10^–8^ M BPA on SR Ca^2+^ release and reuptake were near or at saturation (Figures 2 and 3), whereas BPA only inhibited I_CaL_ by 11% (Figure 4). At 2 × 10^–7^ M, the dose of nifedipine used in our experiments, nifedipine blocked I_CaL_ by 39% (Figure 6A,B), which is consistent with the reported median inhibitory concentration (IC_50_) of nifedipine of 0.3 μM (Shen et al. 2000). We examined the dose response of BPA over the dose range of 10^–12^ to 10^–8^ M as well as 10^–8^ M BPA plus nifedipine. Nifedipine plus 10^–8^ M BPA reproduced the inverted U-shaped dose responses as measured by both myocyte contractility (Figure 6C,D) and incidence of triggered activities (Figure 6E,F). Thus, the monotonic and stimulatory effects of BPA on SR Ca^2+^ release and reuptake, plus the inhibitory effect of higher-dose BPA on I_CaL_, are sufficient to produce the nonmonotonic dose responses of BPA in female rat cardiac myocytes.

## Discussion

Defining the nonmonotonic dose responses of EDCs is of both scientific interest and relevance to understanding the potential health impacts of these chemicals. We show that acute exposure to low doses of BPA (≤ 10^–8^ M) had significant impact on arrhythmogenesis and the mechanics of female cardiac myocytes, and that the dose responses of the rapid impact of BPA are nonmonotonic. The nonmonotonicity is produced by multiple monotonic effects on individual cellular Ca^2+^ handling processes through ERβ-mediated signaling (Figure 6G). Our results add to a growing body of evidence demonstrating the nonmonotonic responses of EDCs and provide mechanistic insights into the pharmacodynamics of BPA’s actions.

Nonmonotonic dose responses of EDCs and hormones have been well documented and are likely a common phenomenon of EDCs (Vandenberg et al. 2012). Nevertheless, their existence and significance have been disputed (Rhomberg and Goodman 2012), citing the lack of statistical significance in some studies and attributing the observations to random dose-by-dose fluctuations and high signal-to-noise ratio. In addition, existing evidence of nonmonotonicity has been faulted for involving only quantitative continuous end points and lacking all-or-none biological events. The lack of mechanistic understanding of the link between quantitatively continuous effects of EDCs and changes in incidence rates of distinct diseases is viewed as a weakness. We assessed the dose response of BPA in cardiac cells using multiple end points, including myocyte contraction, Ca^2+^ dynamics, and arrhythmogenesis, using separate and independent measurement assays; each produced dose responses with marked nonmonotonicity. The response measured at 10^–6^ M BPA for each of the end points showed clear and statistically significant (*p* < 0.01) declines compared with 10^–8^ M; 10^–6^ M BPA was not statistically different (*p* > 0.2) compared with control (Figure 1). Given the consistency among multiple end points, statistical significance of the results, and reproducibility of these nonmonotonic events from previous studies (Yan et al. 2011), we consider it unlikely that the observed nonmonotonic dose responses are attributable to random fluctuation. Of particular significance is the arrhythmogenesis end point (i.e., incidence of triggered activity). Triggered activities are aberrant spontaneous excitations of cardiac myocytes and are well-recognized as one of the key arrhythmogenic mechanisms in the heart (Pogwizd and Bers 2004); therefore, they could be considered a toxicologically relevant end point. The presence or absence of triggered activity is clearly an all-or-none event and was measured as such in our study. The pronounced inverted U-shape of BPA’s effect on this all-or-none end point is evident. Other examples of BPA impacting all-or-none events with nonmonotonic dose responses include the presence or absence of tumor and metastases (e.g., Jenkins et al. 2011). Thus, the nonmonotonic dose response of BPA is not limited to quantitative continuous end points.

Growing evidence has provided increasing understanding of the mechanisms that generate the nonmonotonic dose response of EDCs. The known mechanisms are diverse and have been reviewed in a number of articles (Myers et al. 2009; Vandenberg et al. 2012; Watson et al. 2010). Examples of these mechanisms include actions of different types of receptors (e.g., ERα and ERβ) with opposing signaling effects, cell subpopulation-specific and opposite responses to hormone actions, cytotoxicity associated with higher hormone doses resulting in decreased responses, signaling through parallel pathways with different temporal activation patterns, receptor desensitization, and receptor down-regulation at higher doses. A range of other xenobiotics are capable of producing nonmonotonic effects due to nonspecific mechanisms of action. Here, we describe a distinct mechanism in cardiac myocytes that involves signaling of a single receptor, ERβ, that results in multiple monotonic effects on individual elements of the myocyte Ca^2+^ handling process (Figure 6G). Previously, we showed that the rapid effect of BPA on cardiac arrhythmogenesis and mechanics is mediated by its impact on myocyte Ca^2+^ handling; in particular, increased diastolic SR leak plays a key role in the arrhythmogenic effect of BPA (Yan et al. 2011). The present results show that BPA rapidly increased SR Ca^2+^ release/leak and SR reuptake with monotonic dose responses. Opposing this stimulatory effect is the monotonic suppression of I_CaL_ at micromolar doses. Based on known mechanisms of cardiac Ca^2+^ handling, suppression of I_CaL_ also reduces Ca^2+^-induced Ca^2+^ release from the SR, resulting in reduced Ca^2+^ transient amplitude and myocyte contraction. Suppression of Ca^2+^ influx through I_CaL_ may reduce intracellular Ca^2+^, thereby reducing the development of triggered activities, particularly delayed after depolarizations (Pogwizd and Bers 2004). The exact impact of I_CaL_ inhibition in the presence of enhanced SR Ca^2+^ cycling is complex and influenced by feedback regulatory mechanisms (Eisner et al. 2013). To test the role of I_CaL_ suppression in generating the decline phase of the inverted U-shaped dose–response curve, we observed that mimicking BPA’s suppression of I_CaL_ with the Ca^2+^ channel blocker nifedipine, at a dose that produces a percentage blockade of I_CaL_ similar to that of high-dose BPA, reproduced the inverted U-shaped curve. We do recognize that the result of this experiment is confounded by the fact that we used 10^–8^ M BPA (which produces a 10% blockade of I_CaL_) plus nifedipine to mimic the effect of micromolar BPA, and that the overlapping effects of BPA and nifedipine on I_CaL_ do not fully reproduce the effect of micromolar BPA. Nevertheless, the result qualitatively demonstrates that the nonmonotonic effect of BPA can be accounted in part by I_CaL_ inhibition.

Suppression of I_CaL_ by high-dose BPA is similar to that observed in response to high concentrations of 17β-estradiol (E_2_), suggesting the possibility of a common mechanism. Several studies have shown that supraphysiological E_2_ (10–30 μM) partially suppresses I_CaL_ in cardiac myocytes in multiple speci, including rat, human, and guinea pig (Berger et al. 1997; Kurokawa et al. 2008; Meyer et al. 1998; Nakajima et al. 1999). E_2_ has been shown to suppress I_CaL_ in guinea pig ventricular myocytes, with an IC_50_ of 29.5 nM and a maximum suppression of 40% (Kurokawa et al. 2008), values that are remarkably similar to the dose response for the blockade by BPA. Such suppression of I_CaL_ by high doses of estrogens, while of limited physiological relevance, may play a role in determining the dose–response properties of other estrogenic chemicals in the heart.

It has been shown both experimentally and computationally that nonmonotonic dose responses can be generated by the opposing actions of multiple receptors (Conolly and Lutz 2004; Vandenberg et al. 2012; Watson et al. 2010). In previous studies we found that ERα and ERβ had opposing actions in cardiac myocytes (ERα rapid signaling had an inhibitory effect, whereas ERβ was stimulatory); thus, the rapid actions of BPA in female hearts are mediated by the stimulatory signaling of ERβ, and the counterbalance of ERα versus ERβ results in the lack of observable response to BPA in male hearts (Belcher et al. 2012). Indeed, we speculated previously that the opposing actions of ERα and ERβ generate the nonmonotonic dose response of BPA in the heart (Belcher et al. 2012). This, however, does not appear to be the case. As shown in Figure 5, pharmacological blockade of ERβ largely abolished the rapid effect of BPA on contractility, whereas blockade of ERα had no detectable effect on the inverted U-shaped dose response. Although the sum of BPA’s effects on multiple Ca^2+^ handling processes is sufficient to account for the observed nonmonotonicity, the potential contribution of other mechanisms, such as receptor desensitization, were not examined in the present study and cannot be ruled out. In addition, the molecular/signaling mechanisms underlying BPA’s impact on the Ca^2+^ handling elements are unknown and remain to be elucidated.

## Conclusion

The rapid effects of BPA on female rat cardiac myocytes were characterized by nonmonotonic dose responses, as measured by multiple end points; the cellular mechanism of BPA’s nonmonotonicity involves monotonic and opposing effects on multiple Ca^2+^ handling processes, all mediated by ERβ signaling. The summation of these parallel effects generated an inverted U-shaped dose response, with the most efficacious dose around 10^–8^ to 10^–9^ M, coinciding with reported human exposure levels (Vandenberg et al. 2010) (Figure 6G).
